# Evaluation of Nucleic Acid Isothermal Amplification Methods for Human Clinical Microbial Infection Detection

**DOI:** 10.3389/fmicb.2017.02211

**Published:** 2017-12-12

**Authors:** Brett E. Etchebarne, Zenggang Li, Robert D. Stedtfeld, Michael C. Nicholas, Maggie R. Williams, Timothy A. Johnson, Tiffany M. Stedtfeld, Tanja Kostic, Walid T. Khalife, James M. Tiedje, Syed A. Hashsham, Mary J. Hughes

**Affiliations:** ^1^Department of Osteopathic Medical Specialties, Section of Emergency Medicine, College of Osteopathic Medicine, Michigan State University, East Lansing, MI, United States; ^2^Civil and Environmental Engineering, Michigan State University, East Lansing, MI, United States; ^3^The Center for Microbial Ecology, Michigan State University, East Lansing, MI, United States; ^4^Bioresources Unit, Austrian Institute of Technology GmbH, Tulln, Austria; ^5^Department of Microbiology, Sparrow Laboratories, Sparrow Health System, Lansing, MI, United States; ^6^Department of Microbiology and Molecular Genetics, Michigan State University, East Lansing, MI, United States

**Keywords:** clinical pathogen infection, rapid detection, direct amplification, sepsis

## Abstract

Battling infection is a major healthcare objective. Untreated infections can rapidly evolve toward the condition of sepsis in which the body begins to fail and resuscitation becomes critical and tenuous. Identification of infection followed by rapid antimicrobial treatment are primary goals of medical care, but precise identification of offending organisms by current methods is slow and broad spectrum empirical therapy is employed to cover most potential pathogens. Current methods for identification of bacterial pathogens in a clinical setting typically require days of time, or a 4- to 8-h growth phase followed by DNA extraction, purification and PCR-based amplification. We demonstrate rapid (70–120 min) genetic diagnostics methods utilizing loop-mediated isothermal amplification (LAMP) to test for 15 common infection pathogen targets, called the Infection Diagnosis Panel (In-Dx). The method utilizes filtration to rapidly concentrate bacteria in sample matrices with lower bacterial loads and direct LAMP amplification without DNA purification from clinical blood, urine, wound, sputum and stool samples. The In-Dx panel was tested using two methods of detection: (1) real-time thermocycler fluorescent detection of LAMP amplification and (2) visual discrimination of color change in the presence of Eriochrome Black T (EBT) dye following amplification. In total, 239 duplicate samples were collected (31 blood, 122 urine, 73 mucocutaneous wound/swab, 11 sputum and two stool) from 229 prospectively enrolled hospital patients with suspected clinical infection and analyzed both at the hospital and by In-Dx. Sensitivity (Se) of the In-Dx panel targets pathogens from urine samples by In-Dx was 91.1% and specificity (Sp) was 97.3%, with a positive predictive value (PPV) of 53.7% and a negative predictive value (NPV) of 99.7% as compared to clinical microbial detection methods. Sensitivity of detection of the In-Dx panel from mucocutaneous swab samples was 65.5% with a Sp of 99.3%, and a PPV of 84% and NPV of 98% as compared to clinical microbial detection methods. Results indicate the LAMP-based In-Dx panel allows rapid and precise diagnosis of clinical infections by targeted pathogens across multiple culture types for point-of-care utilization.

## Introduction

Rapid and accurate diagnoses, paired with appropriate and effective interventions, are important to stemming the disease process, maintaining economically feasible care and reducing long-term morbidity of infected patients. Sepsis is recognized as a major cause of morbidity and mortality in infected patients and is estimated to occur in 300 cases per 100,000 people per year in the United States and 18 million cases occur per year worldwide (Angus et al., 2001; Johnston et al., 2017). Systematic approaches to early sepsis identification and intervention including timely broad-spectrum antibiotic administration and adequate fluid volume resuscitation have yielded definite improvements in patient outcomes and health care resource utilization. It has been recognized that one of the limiting factors in treatment of sepsis in the hospital setting is the timeliness of pathogen identification and implementation of appropriate antimicrobial therapy (Rivers et al., 2012). A recent review and meta-analysis of mortality of patients presenting to the emergency department and diagnosed with sepsis indicated that immediate antibiotic administration reduced patient mortality by up to 33% (Johnston et al., 2017). The current “gold standard” of sepsis microbial identification is blood culture, which takes between 2 and 5 days for a definitive species identification. Antimicrobial agent susceptibility for the given organism is obtained within this same timeframe. However, in the period it takes for final culture results, administration of broad-spectrum antibiotics is provided ensure organism eradication. Improving upon this method of non-specific antimicrobial administration and lengthy identification period is achieved with fast and precise species identification as well as antibiotic resistance gene identification. Any delay in successful treatment has consequences for the patient. Long-term medical problems include increased health care costs due to difficulty in pathogen eradication, as microbial populations gain strength against future drug administration.

Treatment of systemic microbial infections is complicated by the fact that the use and overuse of antibiotics has led to the rise in antimicrobial resistant pathogens. Bacteria develop resistance to antibiotic treatments by mutating the genes targeted by them or acquisition of resistance genes from other bacteria. Together, these problems are compounded by the development of multi-drug resistant “super bugs” that have increased pathogenicity and drive up patient morbidity, mortality and health care costs. Methicillin-resistant *Staphylococcus aureus* (MRSA) and Vancomycin-resistant Enterococcus (VRE) are just two examples of commonly encountered causes of sepsis that pose difficulty in eradication by antibiotic therapy (Li et al., 2009). By identifying the organism(s) responsible for infection, physicians could potentially tailor a specific antibiotic regimen to the pathogen at hand, facilitating immediate and optimal treatment to achieve early goal-directed therapy of patients on a level not previously achieved, while preserving broad-spectrum antibiotics for cases in which they are crucial for patient recovery.

Traditional clinical microbiology has relied upon culture-based methods to identify clinical pathogens, but microbial species can be identified using non-culture based methods by using modern techniques (PCR or mass spectroscopy). Pathogen identification is simplified by the fact that a small number of pathogens are the cause of sepsis in the majority of septic patients (Supplementary Table 1). Previous attempts at achieving the goal of rapid pathogen identification have been met with moderate success using a multiplex PCR approach; however, this technique is somewhat challenging given the technical expertise and limitations involved with this diagnostic approach (Avolio et al., 2010; Tsalik et al., 2010). Recent publications aimed at rapid diagnostics offer convincing results by using either culture, DNA extraction follow by real-time PCR for specific detection of uropathogens (van der Zee et al., 2016), or labor intensive gas chromatography – mass spectroscopy based diagnostics for respiratory pathogens (van Oort et al., 2017).

In 2000, Notomi and colleagues described a method of DNA amplification without requiring temperature cycling called loop-mediated isothermal amplification (LAMP) (Notomi et al., 2000). LAMP technology dramatically decreases the time to detection of low abundance DNA templates, typically lowering the threshold for identification from approximately 3 h by traditional PCR to less than 30 min using LAMP (Tourlousse et al., 2012).

Briefly, LAMP reactions allow amplification of template at a target temperature between 60 and 65°C utilizing a polymerase enzyme with high strand displacement and replicative activity amplifying two to three sets of DNA primers. LAMP generally employs at least four primers targeting six distinct regions on the gene to maximize specificity. The LAMP reaction produces copious amounts of DNA product, and when carried out with fluorescent or colorimetric analysis the initial target copy number can be determined (Oh et al., 2016). As previously reviewed (Williams et al., 2017), one of the main advantages of LAMP is the potential for amplification with minimal sample preparation. Studies have shown LAMP from positive blood cultures and DNA purification for MRSA (Misawa et al., 2007), and for *Chlamydia trachomatis* from urine after minimal pre-processing (Jevtuševskaja et al., 2016). In our own previous studies, minimal inhibition was observed in blood, saliva, and urine samples spiked with bacterial pathogens (Stedtfeld et al., 2015). Thus, LAMP has high potential in the field of medical diagnostics due to minimal sample processing and rapid DNA amplification. LAMP has also been previously demonstrated for detection of the Human Immunodeficiency Virus, *Mycobacterium tuberculosis*, (Geojith et al., 2011), *Plasmodium falciparum* (Poon et al., 2006), *Bacillus anthracis* (Dugan et al., 2012), *Pseudomonas aeruginosa, Escherichia coli* (Yan et al., 2016), Zika Virus (Wang et al., 2016), *Clostridium difficile* (Johansson et al., 2016), and *Acinetobacter baumanii* (Loo et al., 2016), to name just a few.

The goal of our study was to develop a rapid test for the presence of common infectious pathogens in human clinical samples with minimal sample processing. We targeted microbes more commonly present in Lansing, MI area hospital patients and compared two separate methodologies to determine detection limits with unique genes characteristic of each microbe. In Step 1, clinical pathogen concentrations were estimated using both a fluorescent-based thermocycler unit (Roche LightCycler 96) and a parallel visual discrimination test utilizing EBT Dye-based reaction color change. In Step 2, we analyzed the presence of molecular targets in patient samples using the In-Dx panel against hospital culture methods from prospectively collected clinical patient samples across clinical culture types.

## Results

### Analytical Performance of LAMP PCR

Single gene targets were selected for comparative quantification of pathogens to enable to effectively rule in or rule out the presence of each pathogen (**Table 1**). Primers were designed to be exclusive to one organism of interest except for *mecA* which is a generalized antibiotic resistance gene target with known associations to a number of clinical pathogens including *S. aureus* and *S. epidermidis*, a coagulase-negative *Staphylococcus* species which has been clinically recognized as a commensal skin contaminant, an opportunistic pathogen, and potential microbial gene-transfer reservoir (Becker et al., 2014; Rossi et al., 2017).

**Table 1A:** In-Dx target organisms by genus and species, target primers, NCBI sequence accession code and sensitivity of assays in Colony Forming Units (CFU) × 10ˆ3 per mL of reaction derived from purified DNA, with *in vitro* dilution from urine and blood results with in-Dx LAMP nucleotide primer sequences.

					Purified	Urine	Blood	Blood						
					DNA	detection	detection	detection						
In-Dx	Microbial	Primer	Gene	NCBI	detection	(whole	(whole	(purified						
ID	target	name	Ontology	accession	RT-PCR	cell spike)	cell spike)	DNA spike)	FIP	BIP	LF	LB	F3	B3
1	*Escherichia coli*	pflB	Formate c-acetyl- transferase 1	LT906474.1	50 pg, 22 m	110 × 10ˆ3 CFU/mL, 30 m	400 × 10ˆ3 CFU/3 mL, 14 m	5 pg, 18 m	TCGCCGCTAAAGTGTCCATCGAT GTTGAAGTCCGGACGCA	GCGGCAGTTTTTCAGACCACAGCC TGAACACCCTGTACACC	TCTTCTCTGCAGTATGAGAACGAT	AATGGTCATGTTCGGTTCCG	GGCTTACGCAGCAAGCAATA	GGTCGTACCCTGGTTACCA
2	*Staphylococcus aureus*	LDH1	Lactate dehydrogenase	NC_002951.2	500 fg, 25 m	80 × 10ˆ3 CFU/mL, 30 m	not tested	5 pg, 17 m	TTGTACCAGAACCTATAACACGTTCCA AATCCTGTTGATATTTTAGCGTA	TATTGTTAAGCGAAGCGTTCGAACCA TGTTCACCAATAATTTGAG	ACCAGAGAATTTCCATGTTGCA	TGTTGCGCCACGTAGCG	TGATGGTATTTTCTTGGTAGCT	CCATACTGGTAATTCAGTGTC
3	*mecA*	mecA	Penicillin binding protein 2	AY894415.1	5 pg, 27 m	40 × 10ˆ3 CFU/mL, 38 m	Not tested	5 pg, 17 m	AATGCAGAAAGACCAAAGCATAC ATGCCAATTCCACATTGTTTCG	TGACGCTATGATCCCAATCTAACT ACTACGGTAACATTGATCGC	TTTAAAATCAGAACGT GGTAAAATTTTAGAC	CCACATACCATCTTCTT TAACAAAATTAAATTG	ATCTCATATGCTGTTCCTGTA	AAAAAACGAGTAGATGCTCAA
4	*Enterococcus faecalis*	BckdE1	Branched-chain alpha-keto acid	AF149712.1	5 pg, 22 m	150 × 10ˆ3 CFU/mL, 30 m	Not tested	5 pg, 27 m	GGAAGGTTTACTCGTAACAGAAG ACGAAATCATTGCGCAATTTCAC	CGTTTTCTTAGCTGCAGCACTG GAATTGATGCCGAAATCG	AATAAAGAAGGCAGCGTGATGA	CGCGATCTAAAGGATATAATGAGCG	TGCATCTAAATCAAAAGAGCA	GGAAAAACTAGCCGCTGAA
5	*Enterococcus faecium*	Fms14	LPXTG family cell surface protein	NC_017960.1	5 pg, 18 min	60 × 10ˆ3 CFU/mL, 38 min	Not tested	500 fg, 30 m	GAATGCGCTGGAGTGCTTGAAAC GTAGCGAAGAAAATGAGA	AACGCCAGTTATTCAACGGTATTA GGATAGATATATTCATAGGGGTC	CGATTGGTTCGTCCAGCTTTTCCA	CGTCTTGGGCCAACAGCTACG	CTGATACAGAAGATTCCGCT	CGTTTTCAACTTCTCCGC
6	*Klebsiella pneumoniae*	Khe	Hemolysin gene	AF293352.1	5 pg, 25 m	50 × 10ˆ3 CFU/mL, 30 m	Not tested	5 pg, 36 m	GCTCAATCCCGGCTATGCCGTC GTGTGGACCGAAGAACT	GCGGTTGTACTTCTTGTTGGCCC ATCTGCCACACCTTTCTCA	ATCGTTGGGTTGACCATCCG	AGACGAACTTCCTGCTCGGTGTTAT	ACTTTTTCCGCGGCTTACC	GGGTTTTCCCGCTGGTAC
7	*Streptococcus agalactiae*	CspA2	Cell surface protease	CP016391.1	5 pg, 40 m	500 × 10ˆ3 CFU/mL, 50 m	Not tested	5 pg, 30 m	CAATAGCTTTAACATAAAGGGCTGGG CACAAGTAATGTTTATGAGAGTC	CGTTAAACTCGGTGCTGATAGCAT TATAAGTCGGTCATCGGC	CCAGTTCCTCTTTTTTCATCAGAGA	TAGGTGGAGCTAATGGCTCTT	CTATGGTGTTGCTCCTGAA	AGCGAGTCTAGCCATCTC
8	*Staphylococcus epidermidis*	gehD	Lipase precursor	AF090142.1	5 pg, 17 m	5 × 10ˆ3 CFU/mL, 20 m	Not tested	5 pg, 13 m	GCACCATAATCTACTCTTCCACCTT CAATGTAGGAGCATTTAGCA	GGTCACAAGCGTTATGGCAGATGT ATCTTTTTACCTGGTTCC	GTTCAACAGCACGGTCATAATTG	ACATATGAAGGCATCATGCCTGA	TTACCGAGTTCACGAAGC	ACCCATACTATGTCCAACAAG
9	*Streptococcus pyogenes*	MstA	M protein	AE004092.2	5 pg, 20 m	8 × 10ˆ3 CFU/mL, 25 m	Not tested	5 pg, 14 m	ACGGCAAAGTAATCTTTGTTTTTC GCAATCTAGAAGATGGTGGGC	ACTTGGAAGGTGCAAGCGAAAAATG ATTCCGTTTTCGAGAT	GCATTTTTCACTAGTTTGGTGAACC	GAAAAGAAGATCCACATGCTTGGT	GCTGATGTGATTTTCTATAATGGTA	CAATCAATTGTTTGGCAATGT
10	*Proteus mirabilis*	DiaA-2	Tyrosine decarboxylase	CP004022.1	50 fg, 20 m	20 × 10ˆ3 CFU/mL, 45 m	Not tested	50 pg, 26 m	CAATTTCATCGGGAATATTCCCCT CGATTAAGCCGTTTTCTCGC	TCCAATCAGCACCTGCAGTTGC GCATTGTCCATACCCAA	TTCTGGGCACAGTTTTGGCGG	GGGGGATCCATCAGCTATATGCTTT	CCTACGGGGATTGTTTGT	AGCTTCCCATTGATTTTGAG
11	*Pseudomonas aeruginosa*	ArcC	Carbamate kinase	NC_002516.2	5 pg, 15 m	30 × 10ˆ3 CFU/mL, 25 m	Not tested	50 p, 24 m	AACTTGTCGCCGTCCGGGGGA AGAAGCCGAGCGCCTG	GAAGCGGATCTTCGAGATCCGCA GATGACGATGGTGCCTTTC	GATGCTCCAGCCCTTCTCG	CCGGTGAAGTGGCTGCT	ATCGGCCCGGTCTACTCC	TCGTACATGGTCGGGATACC
12	*Candida albicans*	PMA1	Adenosine triphosphatase	NW_139471.1	50 pg, 18 m	40 × 10ˆ6 CFU/mL, 20 m	Not tested	5 pg, 17 m	GACATCTTCTGGGATTGGGTGGG AAAGAATTATTTGTGTTAAGGGTG	TTGCCGAATTTGCTTCCAGACCA AAATTTCCCAATGACCTT	ACAGTCTTTAAGACGAATAATGGGG	TCTTTGGGTGTTGCCAGAAAGAGAG	AGTTACTGCTATTGTTGAATCAC	TGGATCCATACATGGCATAA
13	*Enterococcus casseliflavus*	VanC2	D-alanine–D-serine ligase	NG_048353.1	50 pg, 22 m	6 × 10ˆ3 CFU/mL, 32 m	Not tested	5 pg, 18 m	CATAGGGAGATGATTGGAGTGCA CGATACCCGTTTCTTTAGC	ATAGACCTCTCTTTGATCGGGAT TTGTCGGATGTTTTCAGTTC	TTCGATGCGCTAGTTGCTGA	CCCCCAGATGCTATGGATTGGTATT	CGCATATTTTTGGAGGCAATT	CGTATCCAACACCAGTGTC
14	*Enterococcus gallinarum*	VanC1	D-alanine–D-serine ligase	NG_048345.1	5 pg, 25 m	600 × 10ˆ3 CFU/mL, 26 m	Not tested	5 pg, 14 m	GTGGCAGGATCGTTTTCATAGCA TACCATGGGAATCGCTAG	TCGATCGTTTTATTCAAGACCATG GTTGTGATCCCTTTTGAAGAAC	GGGATAAAAGCAAAGTGGGAGCA	ATTCCCGATCTTTATCAAGCCGA	GCTCTTGCATCAACTTGC	AGCGCTGTTTTGTCAGTT
15	*Clostridium difficile*	TdcA	Toxin A	NC_009089.1	Not tested	Not tested	Not tested	Not tested	CCGCCAAAATTTTTTAGGGCTAATA TTTATAGTCAGGAGTTGTTAAATCG	AGATGTTGATATGCTTCCAGGTAT TCTAGTCCAATAGAGCTAGGTC	ATGTCAGATGCTGCAGCTAAATTTC	GAGACTAAATAAATTTTGTTATAG	AGTTTGTTTACAGAACAAGAGTT	ATTTTATCATTTCCCAACGGT

**FIGURE 1:**
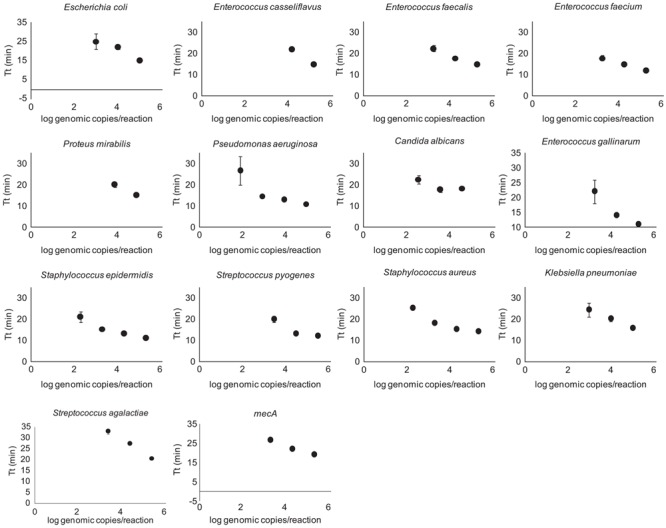
LightCycler Primer validation. Each In-Dx target organism’s detection limit in log genomic copies per reaction well at five minute time intervals using the LightCycler method.

**FIGURE 2:**
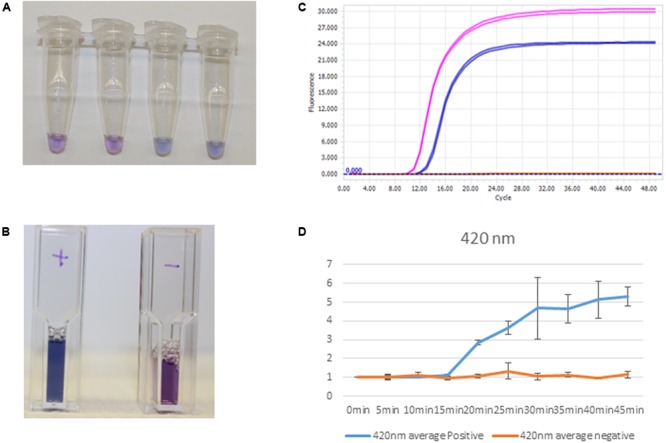
Clinical sample processing and analysis. **(A)** *S. aureus* detection by EBT-color change from purple to blue-Tubes 1 and 2, negative, and Tubes 3 and 4, positive. **(B)** Positives, *S aureus* clinical sample amplification by thermocycler (first, pink tracing) and positive controls, *S aureus* amplification (second, blue tracing). **(C)** Cuvettes with positive (left) and negative (right) color change corresponding to spiked DNA samples. **(D)** Spectrophotometric analysis at 420 nm with corresponding positive control amplification (blue) and negative control (orange) at times 0–45 min for positive and negative samples (*n* = 3).

Quantification of purified genomic DNA from cultured hospital and ATCC sources for the In-Dx pathogens was performed through standard curve generation derived using LightCycler (**Figure 1**) and EBT-based color change analysis (**Figure 2**). Spiked purified DNA in urine and blood was analyzed with LightCycler to estimate primer amplification stability in human derived samples. Spiked whole cultured pathogen cells (except for *C. difficile*) were diluted into urine. Only *E. coli* was spiked into blood, concentrated using EconoSpin and quantified by LAMP analysis (**Table 1B**). Previous studies investigated whole spiked pathogens into blood for LAMP analysis using an alternate platform (Stedtfeld et al., 2015). Primers showing positive amplification from non-target DNA within 50 min were excluded from subsequent tests. Detection thresholds for primer sets using purified DNA ranged from 5 pg at 27 min (*mecA*) to 50 fg at 20 min (*Proteus mirabilis*) by LightCycler analysis (Supplementary Figure 1). Isolates were positive from 5 to 500 pg at 35 min reaction time with EBT-LAMP analysis (**Table 1B**). Color change was detected for the *mecA* primers by EBT-LAMP analysis at 50 pg concentration of purified DNA at 35 min of incubation time (Supplementary Figure 1). No EBT-LAMP samples were incubated greater than 35 min to avoid non-specific primer amplification.

**Table 1B:** In-Dx targets organisms with sensitivity of assays in Colony Forming Units (CFU) × 10ˆ3 per mL of reaction derived from purified DNA, with *in vitro* dilution from urine and blood results for three technical replicates per target organism.

		Purified DNA	Urine detection	Blood detection	Blood detection	EBT test sensitivity
In-DxID	Microbial target	detection RT-PCR	(whole cell spike)	(whole cell spike, EDTA tube)	(purified DNA spike)	35 min (purified DNA)
1	*Escherichia colt*	50 pg, 22 rri	110 × 10ˆ3 CFU/mL, 30 m	5,O00 × l0ˆ3CFU/mL,18 min	5 pg, 18 m	50 pg
2	*Staphylococcus aureus*	500 fg, 25 m	80 × 10ˆ3 CFU/mL,30 m	l,4O0 × l0ˆ3CFU/mL,25 min	5 pg, 17 m	5 pg
3	*mecA*	5 pg, 27 m	40 × lOˆ3CFU/mL,38 m	1,530 × 10ˆ3 CFU/mL (2/3), 40 min	5 pg, 17 m	50 pg
4	*Enterococcusfaecatis*	5 pg, *22* m	150 × 10ˆ3 CFU/mL, 30 m	30,500 × 10ˆ3 CFU/mL, 28 min	5 pg, 27 m	5 pg
5	*Enterococcus faectum*	5 pg, 18 min	60 × 10ˆ3 CFU/mL,38 min	6,000 × 10ˆ3CFU/mL, 23 min	500 fa 30 m	500 pg
6	*Klebsiella pneumoniae*	5 pg, 25 m	5O × l0ˆ3CFU/mL,30 m	900 × 10ˆ3 CFU/mL, 32 min	5 pg, 36 m	50 pg
7	*Streptococcus agatactiae*	5 pg, 40 m	500 × 10ˆ3 CFU/mL, 50 m	430 × lOˆ3CFU/mL,30 min	5 pg, 30 m	5 pg
8	*Staphylococcus eptdermtdis*	5 pg, 17 m	5 × 10ˆ3 CFU/mL, 20 m	1,700 × lOˆ3CFU/mL,20 min	5 pg, 13 m	50 pg
9	*Streptococcus pyogenes*	5 pg, 20 m	8 × l0ˆ3CFU/mL,25 m	1 × l0ˆ3CFU/mL,18 min	5 pg, 14 m	50 pg
10	*Proteus mirabills*	50 fg,20 m	20 × 10ˆ3 CFU/mL, 45 m	260 × 10ˆ3 CFU/mL, 36 min	50 pg, 26 m	50 pg
11	*Pseudamanas aeruginosa*	5 pg, 15 m	30 × 10ˆ3 CFU/mL, 25 m	15,00O × l0ˆ3CFU/mL,18 min	50 p, 24 m	50 pg
12	*Candida albicans*	50 pg, 18 m	40 × 10ˆ6 CFU/mL, 20 m	3,100 × 10ˆ3 CFU/mLI 1/3), I8 min	5 pg, 17 m	50 pg
13	*Enteracoccus cassetlftavus*	5O pg, 22 m	6 × 10ˆ3CFU/mL, 32 m	3,900 × 10ˆ3 CFU/mL, 24 min	5 pg, 18 m	50 pg
14	*Enteracaccus gallinarum*	5 pg, 25 m	600 × 1Oˆ3CFU/mL, 26 m	2,000 × 10ˆ3 CFU/mL, 18 min	5 pg, 14 m	50 pg
15	*Clostridium difficile*	Not tested	Not tested		Not tested	Not tested

### Clinical Detection by In-Dx

In total, 239 samples were collected across culture types (31 blood, 122 urine, 73 mucocutaneous wound/swab, 11 sputum and two stool samples) from 229 consecutive prospectively enrolled patients with suspected clinical infection with samples analyzed both at the hospital and by In-Dx (**Table 2**). Nine patients had two sample types analyzed by the In-Dx panel. Across blood, urine, wound/swab, sputum and stool samples the overall sensitivity of the In-Dx panel for detection of the target pathogens from 239 clinical samples was 76% with a specificity of 98%. The positive predictive value for all samples was 64% and negative predictive value was 99% (Supplementary Appendix 2B).

**Table 2:** In-Dx versus hospital culture results by culture type.

Culture type	Samples (*n*)	True positive	True negative	False positive	False negative	Sensitivity	Specificity	PPV	NPV
Blood	31	1	429	1	3	25	99.8	50	99.3
Urine	122	51	1608	44	5	91.1	97.3	53.7	99.7
Swab	73	38	957	7	20	65.6	99.3	84.4	98
Sputum	11	3	148	2	1	75	98.7	60	99.3
Stool	2	1	1	0	0	100	100	100	100
All	239	94	3143	54	29	76.4	98.3	63.5	99.1

*A summary of data for performance of the In-Dx panel across culture types for the 15 In-Dx targets. Samples indicate total prospectively matched samples within each sample type. True positive indicates equivalent positive hospital and In-Dx results. True negative indicates equivalent negative results. False positive indicates negative by hospital culture and positive by In-Dx. False negative indicates positive by hospital culture and negative by In-Dx. Sensitivity, specificity, and positive and negative predictive values are expressed as percentages as described in section “Statistical Analysis.”*

Out of 31 matched blood cultures analyzed, hospital culture methods detected *E. coli* twice (In-Dx concordant detection in one of these two samples), MRSA once (no In-Dx detection), and one of two clinical blood culture samples taken from the same patient was positive for methicillin sensitive *S. epidermidis* (In-Dx negative). *E. coli* was detected from one sample by In-Dx for which one of two hospital blood cultures came back positive for unnamed micrococcus species. Sensitivity for positive detection of the 14 targets from blood using the filtration methodology was 25% (*n* = 1/4). Negative predictive value was 99.3% as three positive cultures were called negative by the In-Dx method used. It is very likely that the micrococcus and *S. epidermidis* positive cultures, both positive for only one of two blood collection tubes, are contaminants of little clinical value (Supplementary Appendix 1).

For urine sample testing, 122 duplicate clinical samples were tested for presence of 15 molecular targets. 51 targets were equivalently detected by hospital and In-Dx across these matching culture samples. Negative agreement was present for 1715 tests (including *mecA* identification) (**Table 3**). Overall sensitivity of the In-Dx panel for urine pathogen detection was 91% and specificity was 97%, with a positive predictive value of 54% and a negative predictive value of 99.7%. The In-Dx panel identified six target pathogens corresponding to hospital culture samples resulted as “normal flora” (**Table 3**). In-Dx methods detected an additional 38 pathogen targets from clinical urine samples that were not positive by hospital cultures. Eight of these were *S. epidermidis* (C_t_ range 16–35 (Supplementary Appendix 1), four with an associated positive *mecA* amplification. Another eight of these additional positives by In-Dx testing were *E. coli* (C_t_ range 10–28). *E. coli* was the most common uropathogen detected by both hospital culture (*n* = 36) and In-Dx methods (*n* = 48). Overall sensitivity for *E. coli* by In-Dx was 95% and Sp was 90%. *E. coli* identification by In-Dx was positive for an alternate pathogen by hospital culture results not targeted by the In-Dx panel four times (*Citrobacter koseri, Enterobacter cloacae, Candida* species (non-*albicans* non-*gabralta*), and *Klebsiella ornitholytica* one time each). The In-Dx did not identify a pathogen by direct In-Dx amplification that was found by hospital urine culture five times: twice for *E. coli*, and once each for *E. faecalis, E. faecium* and *S. agalactiae*. Each of these was confirmed positive by In-Dx from cultures grown from residual reserved clinical sample. In-Dx cycles to threshold positive ranged from 9 to 28 min for samples with concordant positive results and >100,000 CFU/mL reported concentration (Supplementary Appendix 2A).

**Table 3:** Urinalysis comparison In-Dx versus hospital culture results by organism.

Target result	+/+ (TP)	+/- (FP)	-/+ (FN)	+/0 (FP)
*Escherichia coli*	36	8	2	2
*Staphylococcus aureus*	1	1		
*mecA*		8		1
*Enterococcus faecalis*	5	5	1	2
*Enterococcus faecium*	1	1	1	
*Klebsiella pneumoniae*	1	3		
*Streptococcus agalactiae*		1	1	
*Staphylococcus epidermidis*	1	8		
*Streptococcus pyogenes*				
*Proteus mirabilis*	1			1
*Pseudomonas aeruginosa*	3	1		
*Candida albicans*	2	1		
*Enterococcus casseliflavus*				
*Enterococcus gallinarum*		1		
**Sum**	**51**	**38**	**5**	**6**

*Urinalysis comparison, hospital culture results versus In-Dx thermocycler analysis: Column 1, + In-Dx/+ HCR 1-14, indicates true positive (FP) equivalent identification by both In-Dx panel and by hospital culture results for the 13 urinary tract infection species targets as well as *mecA* Column 2, + In-Dx / -HCR 1-14, indicates false positive (FP) clinical samples identified as positive by In-Dx and negative by hospital culture result. Column 3 indicates false negative (FN) with negative result by In-Dx and positive by hospital culture and Column 4 shows false positive (FP) samples found positive by In-Dx and called “Normal Flora” by hospital.*

Among 73 duplicate wound and throat swab samples analyzed, sensitivity for presence of targets was 65.5% and specificity was 99.3% (**Table 4**). *S. aureus* was the most frequently detected pathogen among wound and swab samples (*n* = 25 by hospital culture) and sensitivity for *S. aureus* detection by In-Dx methods was 68% with a specificity of 99%. Presence of *mecA* was found in association with detection of *S. aureus* and *S. epidermidis* as well as untargeted *Citrobacter koseri* (*n* = 1) and unidentified background flora. Targets missed by In-Dx by mucocutaneous swab analysis included *S. aureus* (*n* = 8), *mecA* (*n* = 4), *E. faecalis* (*n* = 2) and *S. agalactiae* (*n* = 2), *E. coli, K. pneumoniae, S. pyogenes* and *C. albicans* (*n* = 1 each) (**Table 4**). In-Dx detected methicillin-resistant *S. epidermidis* in two samples which were not identified by hospital culture methods. Hospital methods identified three samples as “normal flora” but positive by In-Dx for *S. aureus* and *E. faecalis* once each (**Table 4**).

**Table 4:** Mucocutaneous swab culture results comparison by organism.

Target result	+/+ (TP)	+/- (FP)	-/+ (FN)	+/0 (FP)
*Escherichia coli*	2	1	1	
*Staphylococcus aureus*	17		8	1
methicillin resistance	6	2	4	
*Enterococcus faecalis*			2	1
*Enterococcus faecium*				
*Klebsiella pneumoniae*			1	
*Streptococcus agalactiae*	5		2	
*Staphylococcus epidermidis*	1	2		
*Streptococcus pyogenes*	4		1	
*Proteus mirabilis*	3			
*Pseudomonas aeruginosa*				
*Candida albicans*			1	
*Enterococcus casseliflavus*				
*Enterococcus gallinarum*				
**Sum**	**38**	**5**	**20**	**2**

*Mucocutaneous swab comparison, hospital culture results versus In-Dx thermocycler analysis across targets: Column 1, + In-Dx = + HCR 1-14, indicates true positive (TP) equivalent identification by both In-Dx panel and by hospital culture results for the 13 mucocutaneous infection species targets as well as *mecA* by target. Column 2, + In-Dx / -HCR 1-14, indicates false positive (FP) clinical samples with positive result by In-Dx and negative by hospital culture result. Column 3 indicates false negative (FN) results for In-Dx with positive result by hospital culture and negative by In-Dx. Column 4 shows false positive (FP) samples with positive results by In-Dx and identification as “Normal Flora” by hospital methods.*

Eleven sputum samples were analyzed using the In-Dx panel. Pathogenic concentrations of *E. coli* (*n* = 1), *P. aeruginosa* (*n* = 1) and MRSA (*n* = 3) were detected by the panel. Of these, samples, agreement was reached for MRSA (*n* = 3) and *E. coli* (*n* = 1). In-Dx identified *P. aeruginosa* and *E. coli* one time each where hospital culture were called negative and normal flora, respectively. In-Dx did not detect *S. aureus* in one sputum sample identified as positive by hospital sputum culture methods. Negative agreement was reached for nine samples (Supplementary Appendix 2A).

Only two stool samples were studied using direct amplification methods for detection of *C. difficile* (Supplementary Appendix 2A). *C. difficile* toxin was identified by both direct amplification methods and hospital *C. difficile* toxin screening methods for one of these samples. Additional gastrointestinal pathogens can be added to help guide clinical decision making for infectious diarrheal complaints.

Color change detection by EBT-LAMP was tested as a secondary direct amplification testing technique for 12 clinical samples included in this study (8 urine samples, 3 mucocutaneous swabs and 1 stool sample tested for *C. difficile*). Eleven of these were confirmed positives by hospital testing including the stool sample positive for *C. difficile* toxin by hospital testing. None of the samples was *mecA* positive by hospital testing or EBT-LAMP analysis (Supplementary Appendix 2A).

### Sample Processing Time

All LAMP reactions were completed within 120 min from start to finish per clinical sample. Time savings was gained when multiple samples were processed simultaneously in batches of up to three 96-well plates. Initial processing time was focused on filtration of blood, urine through EconoSpin spin Column filter tubes (∼10 min) and collection of sample from mucocutaneous swab tubes (∼3 min) followed by heat lysis at 95°C for 15 min. The lysed sample is then mixed with isothermal reaction reagents and pipetted onto 96-well plates preloaded with target primers, sealed and analyzed by either thermocycler (45 min run time with isothermal amplification) or visually (35 min isothermal amplification).

## Discussion

### Diagnosing Pathogenic Infection in Humans

Infection is one of the greatest problems faced by humanity and directly impacts care provided from every healthcare field. Care for the critically infected accounts for an enormous amount of health care resource expenditure. Every minute passing with untreated clinical infection can be reasonably expected to increase the total morbidity and mortality of the infected patient, while increasing the potential strength and pathogenicity of the infecting organism(s) as it survives, proliferates and spreads to other hosts while untreated. While the human body can overcome infection from most potential microbial invaders, those who are already sick or immunocompromised can fall victim to infection. Infections by dangerous pathogens are difficult to eliminate without proper antimicrobial therapy and spread readily among us. The length of time required for standard hospital testing of most microbial pathogens limits accurate and effective diagnosis and treatment of infection.

Methodologies for direct amplification of DNA and RNA sequences to target genetic regions of interest can allow rapid discrimination of microbial pathogens in a point-of-care time-frame. Utilizing either fluorescence detection by a real-time PCR instrument or naked-eye visual detection of color change from purple to blue using EBT-dye chelation, the direct amplification methods used here offer diagnostic capabilities equivalent to or improved compared to the gold standards of clinical microbial pathogen identification, but in a significantly reduced time frame and with lower resource use.

Our results provide evidence that direct amplification methodologies can overcome many limitations of detection of infectious pathogens. We have shown that low but clinically relevant pathogen loads do not appear to limit detection of pathogens directly from urine, wound, sputum and stool samples. Relatively high sensitivities were found for the pathogens targeted by In-Dx, especially with urine samples for which 3 mL of the clinical urine sample was used per panel run.

### In-Dx Identifies of Pathogens across Human Sample Types

Our methods are robust yet sensitive to pathogen concentrations from samples across a diverse set of tissue types. The organisms targeted by In-Dx account for >70% of positive clinical blood and >85% of positive urine culture results in 2013 (Supplementary Table 1). Sufficient clinical sample (>5 mL) is available from most urinalysis specimens for a great deal of molecular testing. Positive identification is clear from most samples after 20 min by isothermal amplification. Strongly positive clinical samples with >100,000 CFU/mL by culture averaged a Ct value of 14.4 min by LightCycler analysis (*n* = 39). It does not seem likely by these results that false positive of clinical pathogens by In-Dx is a problem. The 91% overall sensitivity of the In-Dx panel was higher for urine samples when compared to culture results from two different hospital institutions. False negative detection by In-Dx was low for the targeted organisms as compared to hospital were rare compared to hospital culture (5/121) (Supplementary Appendix 2A). Reasons for failure of these samples are unknown but one (Pt ID 222) was possibly are due to reaction error as subsequent repeat testing from culture was positive for *E. faecalis* as indicated by hospital culture. Cultures did not show growth for other false negative samples.

Although a low sensitivity (25%) was present for blood samples, the clinical sample size is too low to make confident predictions about utility of the process used here for direct amplification. Many of our clinical samples were collected from patients with stable vital signs and of a mild to moderate state of illness. *In vitro* testing suggests that Purple Top EDTA Blood Collection tubes are best for analysis using In-Dx methods. A low abundance of target template is expected from the small amount of blood sampled for direct amplification analysis. LAMP methods have been used successfully to identify pathogens from blood that is already pre-cultured and shows signs of colony growth. No studies appear to have been published to date in which direct amplification from whole blood at the point-of-care is performed. We are currently working to lower our threshold for identification with alternate processing methods to enable detection of bacteremia.

More critically ill patients would reasonably be expected to carry a higher concentration of pathogens per mL of circulating blood. The median Glasgow Coma Score among patients was 15 (out of 15) with an average shock index (heart rate/ systolic blood pressure) of 0.71, an average mean arterial pressure (diastolic blood pressure + 1/3 (systolic blood pressure – diastolic blood pressure)) of 99 and an average lactic acid concentration of 1.68 mg/dl. Therefore, a low percentage of these patients would be likely to have significant bacteremia. A larger sample size from a cohort of more critically septic patients will likely improve results of direct blood testing by the In-Dx method.

The sensitivity threshold and range displayed by our direct amplification methods appears to correlate much more strongly with “moderate” or “many” laboratory reported results for wound and sputum samples, and to >50,000 CFU/mL for urine output results. Outputs from clinical urine samples from Sparrow Hospital are presented in concentration ranges from 10,000 to 25,000, 25,000 to 50,000, 50,000 to 100,000 and >100,000 CFU/mL. *E. coli* was not detected twice from urine (once at a concentration of 10,000–25,000 CFU/mL, once >100,000 CFU/mL. 10,000–25,000 CFU/mL may be below the limit of detection for the direct amplification method for urine, at least for this probe set, though the 21 additional pathogens identified by In-Dx testing and subsequently confirmed in culture argue toward potential under-detection by hospital clinical methods.

### Specific Primer Sets Reveal Specific Pathogens

Results from direct amplification testing of sputum samples show great promise for LAMP methods to rapidly reveal potential infectious respiratory pathogens including *P. aeruginosa*. A panel with inclusion of more common respiratory pathogens will provide a valuable infection diagnostic tool. More samples are needed to fully address detection capabilities for sputum samples.

*Staphylococcus aureus* and MRSA were the most under-detected clinical targets. These pathogens are of the highest clinical abundance and were missed on first pass POC testing about 30% of the time. Lactate dehydrogenase was chosen due to its relative low homology to all other known genetic sequences explored *in silico* and presumptive high abundance of mRNA and DNA for amplification given its core metabolic genetic function. It is noted that *mecA* detection lags approximately 4 min in each case likely reflecting lower marker abundance relative to LDH. This apparent decreased sensitivity translates to further under-detection of *mecA* by EBT color changed analysis from samples found to be positive by LightCycler analysis. No detection of mecA has been noted on a MRSA sample with the EBT method in this study. Sensitivity *in vitro* shows detection of 600 pg purified DNA per reaction at 35 min with the EBT method.

Numerous reasons may account for under-detection of wound swabs. In most wound swab samples collected the duplicate second, third or even fourth swab was sent for analysis by In-Dx. Clinical sample analysis preference was always given toward hospital testing to avoid potential underdiagnoses of patient infection. Often, the duplicate sample for In-Dx analysis had a very small amount of clinical sample to be analyzed as a result of decreased sample availability following replicate collections.

The amount of clinical swab saturation with sample appears to be directly correlated with direct amplification findings for positive samples. Although very little sample is needed, limits are certainly present for detection. All hospital clinical *S. aureus* and MRSA sample positives missed by the first pass using In-Dx were retested on cultures grown in trypticase soy broth and were found to confirm hospital culture findings. All clinical samples were grown in culture and those with positive growth in trypticase soy broth were retained and frozen for retesting. It is also possible that the lower sensitivity of detection from mucocutaneous swab samples is due to interfering substances present with the clinical sample. To determine the influence of interfering factors on reactions an internal control PCR set should be included in future studies.

Advantages to the rapid diagnostic methods employed here include amelioration of appropriate antimicrobial strategy selections including selection of antibiotics with a high degree of specificity for eradication of infection. For example, *E. coli* was 97% sensitive to nitrofurantoin and 89–97% sensitive to cephalosporin antibiotics versus lower sensitivities for more commonly prescribed empiric urinary tract infection antimicrobials such as trimethoprim/sulfamethoxazole (78%) and fluoroquinolones (79–80%) (Supplementary Table 2: Sparrow and McLaren of Greater Lansing Hospital Antibiotic Sensitivity Summary 2015). Monomicrobial pathogenic infections by *E. coli* accounted for 48.96% of all urinary tract infections (*n* = 9714) and 9.56% (*n* = 261) of bloodstream infections in through Sparrow Hospital clinical laboratory testing (Supplementary Table 1). Similar precision with antibiotic prescription selection would be much easier to enact based on the results seen with direct amplification. As the burden of antibiotic resistance transmission increases, particularly within strains of enterobacteriaceae, precise antibiotic prescription should be of increasing importance in antimicrobial therapy decision making.

Polymicrobial detection from clinically infected sources is equal or greater to that of protracted culture methods. Positive polymicrobial cultures from clinical wound samples often revealed microbes of questionable clinical relevance including *Corynebacterium* species, micrococcus species, and peptostreptococcus species, all of which are likely non-pathogenic normal flora. General microscopic identification results with Gram stain and cell morphology but without genus and species designation assigned as final result outputs are generally presented without associated antibiotic sensitivity and resistance results. This begs the question of clinical relevance, especially when reported concentrations are few, rare or moderate after 4–6 days of culture and modification of antibiotic therapy will continue to be empirical at best.

EBT-based color change reactions offer the strengths of detection by direct amplification without the potential limitations present with advanced electronics utilized with a thermocycler based platform. Reactions are performed with a heating block capable of reaching isothermal amplification temperatures, an ice bath, and an individual who can detect color change from purple to blue. Limitations include longer incubation time to detect low abundance targets (such as resistance genes including *mecA*) when clinical sample volume is limited. A longer amplification time (35-45 min) can be used on a separate plate to identify lower abundance genes. To prevent non-specific amplification from species-specific metabolic genes and increase chances of false positive identification all targets running concurrently must amplify in the same timeframe for naked eye detection of color change. When samples are pre-cultured for increased target abundance, such template concentration-restricted scarcity can be overcome.

Our methods offer advantages in speed, sensitivity, specificity, scalability, flexibility in target selection, and conservation of resource utilization. Limitations revealed in the sample population studied include low sensitivity for bloodstream infection. This hindrance is due to a low number of samples studied, low positive rate among the samples studied, variability in the types of samples received (included were EDTA and Bactec aerobic culture bottles). Additionally, only 3 mL of blood was processed to test against the 14 targets. Processing a larger volume of blood for In-Dx could aid in clearing the threshold for pathogen detection directly from these samples for potential point-of-care blood testing. Alternative methodologies for target extraction from bloodstream including nanoparticles and microbeads and premixing reagents with stabilization of reactions at room temperature will help in ease of processing to increase potential for bedside diagnostics by clinical staff with limited training.

Direct amplification of target nucleic acids utilizing isothermal PCR techniques can be adapted toward direct and rapid processing of clinical samples for accurate detection of the primary pathogens responsible for clinical infection across a wide variety of clinical types at the point-of-care. Further testing on additional samples can elucidate testing limitations and generalizability among the pathogens to allow a paradigm change in clinical microbiological testing and infection surveillance and control.

## Materials and Methods

### Ethics Statement

All experimentation with human samples was conducted on samples taken from consenting patients or their designated representative following the Human Research Protection Program and performed in accordance with institutional regulations after pertinent review and approval by the Institutional Review Board at Michigan State University (East Lansing, MI, United States), Sparrow Hospital (Lansing, MI, United States), and McLaren of Greater Lansing Hospital (Lansing, MI, United States) Investigational Review Boards MSU IRB# C13-033F. Written consent was obtained from healthy individuals that donated blood, urine, and sputum samples. Cultured bacterial isolates used for spike-in controls were determined to be non-human subject research as described in a previous study MSU IRB# 12-706 (Stedtfeld et al., 2015). Patient clinical samples were collected between 02-22-2015 and 06-22-2016.

### Study Design

Candidate isothermal amplification primers were generated from review of literature and through NCBI BLAST analysis of potential candidate gene regions (Altschul et al., 1990). LAMP primers were designed using PrimerExplorer V4 software based on consensus sequences obtained for target genes (Kimura et al., 2011). Sensitivity, specificity and reproducibility for LAMP primers were first evaluated using purified genomic DNA isolated from known cultured strains of target pathogenic microbes with known hospital antibiotic sensitivity and resistance results as previously described (Stedtfeld et al., 2015). The strongest performing primer sets for each target gene were evaluated against unprocessed duplicate clinical culture samples on 96-well plate format using real-time PCR detection by Roche LightCycler 96 System analysis and visual discrimination of color change of reactions in the presence of Eriochrome Black Dye (EBT) after incubation on a 96-well plate-adapted heat block. The LAMP assays for detection of 14 clinical pathogens as well as the *mecA* gene were compared with conventional hospital culture and PCR-based assays for sensitivity and specificity of detection directly from clinical samples obtained in the Emergency Department from consenting patients at two regional hospitals, Sparrow Hospital, a Level One Trauma Center, and McLaren of Greater Lansing Hospital, a Level Two Trauma Center, both in Lansing, MI. Samples were first analyzed using LightCycler amplification and a subset were run using EBT-dye methods. Clinical samples with less than 6 mL of urine or an undetectable signal by LightCycler were excluded from EBT analysis.

### Primer Design and Loop-Mediated Isothermal Amplification with Genomic DNA

A six-primer system was employed for the LAMP reaction detection of clinical pathogens. Primer targets were selected from literature or designed using PrimerExplorer V4^1^ online software. Between two and nine primer sets, including a Forward 3 (F3), Backward 3 (B3), Forward Inner Primer (FIP), Backward Inner Primer (BIP), Loop Forward (LF) and Loop Backward (LB) for a total of six primers per target, were developed for each strain and optimized for sensitivity and specificity. Each primer set was tested with purified genomic DNA to achieve detection of ≥5 pg within 30 min. The most sensitive and specific primer set for each microbial target was selected for clinical sample detection (**Table 1**). For generating standard curves, genomic copies per reaction for each isolate was estimated based on mass of gDNA used per reaction and the average genome size for the respective species.

### Hospital Culture Analysis

Culture identification was performed using Siemens Microscan (Beckman Coulter, Inc.), BD Phoenix Automated Microbiology System (Becton, Dickinson and Company), or biochemical tests. Prior to revival of clinical samples for primer validation, isolates were stored in 15% glycerol stocks at -80°C. Isolates were revived by growing on tryptic soy broth (TSB) media overnight at 37°C (no agitation) and serial diluted in 1× Phosphate Buffered Saline (PBS). Ten microliters of each serial dilution was plated on trypticase soy agar (TSA) plates (in triplicate) and colony forming units (CFU) were counted following 24 h of incubation at 37°C.

Microbial culture samples used for primer validation studies included Methicillin-resistant *S. aureus, S. aureus, Streptococcus agalactiae, Streptococcus pyogenes, Enterococcus faecalis, Enterococcus faecium, Escherichia coli*, Methicillin-resistant *S. epidermidis, Proteus mirabilis, Klebsiella pneumoniae, C. difficile* and *Candida albicans*. Each was a validated clinical culture sample from Sparrow Hospital. *Enterococcus casseliflavus (ATCC: 25788), Enterococcus gallinarum (ATCC: 49673)*, and *Pseudomonas aeruginosa (ATCC: 10145)* are from American Type Culture Collection (ATCC, Manassas, VA, United States).

### DNA Extraction from Microbial Strains for Primer Validation with Thermocycler and Eriochrome Black T Dye

Extraction of DNA from bacterial strains for initial primer testing was performed with a DNeasy Blood & Tissue Kit (Qiagen) according to the manufacturer’s instructions. 1.5 mL of bacteria growth culture was used for each strain. The elution volume was 100 μL and the concentration was finally adjusted to 5 ng/μl. Ten-fold serial dilutions of purified genomic DNA from 500 pg/μL to 5 fg/μL for each strain were used for DNA standard curve control generation (**Figure 1** and Supplementary Figure 1).

Minimum concentration for detection was determined for reactions in the presence of Eriochrome Black T dye for ten-fold serial dilutions of purified genomic DNA from 500 pg/μL to 5 fg/μL for each strain with visual and spectrophotometric analysis at 5 min intervals from 0 to 45 min to determine EBT sensitivity standard curve generation (**Figure 2**).

EBT color measurement was performed by spectrophotometer at the wavelengths 380, 400, 420, and 475 nm. Seventy microliter LAMP reaction systems were employed for this purpose. After the LAMP reaction, 64 μL of reaction mixture was taken to the test cuvettes in which preload with 448 μL of water (eight times dilution). After mixing well, absorbance values of the cuvettes were read by a GENESYS 10 Series spectrophotometers from Spectronic Unicam (**Figure 2**).

### Clinical Specimens

A total of 239 duplicate clinical blood (*n* = 27), urine (*n* = 122), wound/throat swab (*n* = 73), expectorated sputum (*n* = 16), stool (*n* = 2) samples from 229 consecutive consenting Emergency Department patients with suspected infection were collected over a period of 16 months from McLaren of Greater Lansing Hospital and Sparrow Hospital. No cerebrospinal fluid samples met inclusion criteria for this study. All clinical samples used in this study were stored at 4°C immediately after collection until nucleic acid template preparation. The stored samples were processed within 24 h of clinical collection time. Clinical template extracts were applied to LAMP reaction immediately following template preparation. Clinical samples were excluded in cases of suspected or confirmed external contamination and or other sample collection and processing problems. All clinical samples from patients with missing or erroneous consent forms were discarded (Supplementary Appendix).

### Nucleic Acid Template Preparation from Clinical Blood, Urine, Wound, Sputum, and Stool Samples

#### Blood Samples

Three milliliter of blood from each Red-top BD Vacutainer Plus Venous Blood Collection Tube Serum Clot Activator, Purple-top BD Vacutainer K2 EDTA Venous Blood Collection Tube, or BD BACTEC Plus Aerobic/F blood culture solution sample, was taken and passed through an EconoSpin Column for crude DNA extraction. Hundred microliter of water was then added to the column within a collection tube and heated on a heating block at 95°C for 15 min.

#### Urine Samples

Three microliter of whole urine from each clinical sample was passed through an EconoSpin Column for collection of genetic material. Next, 100 μl of water was resuspended on the column filter and heated on a heating block at 95°C for 15 min.

#### Wound/Swab Samples

Hundred microliter of liquid was added and then aspirated from the bottom of a BD BBL CultureSwab EZ tube using 200 μl pipettes and transferred to 1.5 mL Eppendorf tube. Repeated aspirations were needed to extract 100 μl from most tubes. Sample was then heated on a heating block at 95°C for 15 min.

#### Stool and Sputum Samples

Four hundred microliter of sample was transferred directly from clinical sterile hospital collection container to 1.5 mL Eppendorf tube. This sample was then heated on a heating block at 95°C for 15 min.

After heating, 1 μl of template from each clinical sample type was added per 10 μl reaction well in a 96-well plate format and analyzed on by LAMP Thermocycler and Eriochrome Black T (EBT) colorimetric change.

### Thermocycler SYTO-82 Reactions

LAMP primers were pre-dispensed in 96-well plates to result in a final concentration of 1.6 μM FIP primer, 1.6 μM BIP primer, 0.8 μM LF primer, 0.8 μM LB primer, 0.2 μM F3 primer and 0.2 μM B3 primer. One target assay was included per well. LAMP Thermocycler reactions were carried out on Roche LightCycler 96 System in 10 μl volume. The LAMP reaction mixture contained 1X Isothermal Amplification Buffer (New England BioLabs), 6 mM magnesium sulfate, 0.64 Unit/μl Bst 2.0 DNA polymerase (New England BioLabs), 20 μM Syto 82 (Molecular probes/Life Technologies), 0.4% Pluronic F-68, 1 μg/μl Bovine Serum Albumin (BSA), 350 μM of each dNTP (Invitrogen), and 1 μl Template.

All primers were synthesized by Integrated DNA Technologies, Inc. The LAMP LightCycler reactions proceeded at 63°C for 40 min. Each target was tested in triplicate with one positive control and one no-template control (5 wells per target). Targets 1–15 were always included in clinical blood, urine, mucocutaneous swab, and sputum analysis. *C. difficile* was the only target for clinical stool samples (*n* = 2).

### Eriochrome Black T (EBT) Clinical Sample Reactions

A subset of positive clinical samples identified by the LightCycler method was tested in 96-well PCR plate format using the colorimetric color indicator azo dye Eriochrome Black (*n* = 12). EBT is an indicator that causes a color change of solution according to calcium or magnesium concentration. As Mg^2+^ concentration decreases during a LAMP reaction in the presence of EBT, the solution changes from purple to blue. Color change measurements were identified visually and using a spectrophotometer (Genesys 10 UV) with measurements recorded at 5 min intervals from 0 to 45 min at 380, 400, 420, and 475 nm wavelengths. Identical LAMP primers used for LightCycler reactions (1.6 μM FIP primer, 1.6 μM BIP primer, 0.8 μM LF primer, 0.8 μM LB primer, 0.2 μM F3 primer and 0.2 μM B3 primer) were pre-dispensed into 96-well plates, and each 10 μL reaction mixture contained 10X Isothermal Amplification Buffer (New England BioLabs), 6 mM magnesium sulfate, 0.64 Unit/μl Bst 2.0 DNA polymerase (New England BioLabs), Eriochrome Black 75 μM (Sigma Aldrich), 0.4% Pluronic F-68, 1 μg/μl BSA, 350 μM of each dNTP, and 1 μl Template. One target assay was included per well. The LAMP reaction mixture was incubated at 63°C for 40 min on a 96-well block heater (Thermo Scientific Compact Digital Dry Bath/Block Heater) followed by immediate immersion in an ice water bath (to stop the reaction and decrease condensation on clear sealing tape) for 1 min before immediate visual color change discrimination from purple to blue by one or more non-color blind human examiners. Colorimetric detection results remained stable for at least 24 h. As with real-time cycler analysis, each target was tested triplicate with one positive control and one negative no template control (5 wells per target), targets 1–15 were always included in clinical urine, mucocutaneous swab and stool analysis and *C. difficile* was the only target for clinical stool samples.

### Data Analysis

For real-time LAMP analysis, cycles to threshold (C_t_) was calculated from the starting point of the amplification signal curve increased 0.01 (arbitrary units) above the baseline signal calculated by LightCycler default autoanalysis. Amplification of at least two of the triplicate wells per target was needed for determination of a sample as positive. The latest of the triplicate positives for each sample was recorded as the amplification time for this sample. A cutoff of 40 cycles (53 s per cycle) was used to minimize false-positive and non-specific primer amplification. The only exception was for *C. difficile* primers which were found to amplify later. The cutoff for the *C. difficile* primer set was set to 40 min (Supplementary Figure 1).

For EBT dye analysis, spectrophotometric data was recorded by readings for positive and negative samples at 5 min intervals from 0 to 45 min at wavelengths 380, 400, 420, and 475 nm. Amplification of at least two of the triplicate wells per sample was used for determination of positive. The cutoff of 45 cycles was used to minimize false-positive and non-specific primer amplification (Supplementary Figure 1).

### Statistical Analysis

Clinical data corresponding to duplicate samples obtained for LAMP analysis were abstracted by retrospective chart review. Patient characteristics and laboratory values, including hospital culture results by organism isolated across culture types, were recorded. All clinical samples with hospital culture or equivalent testing and with a corresponding duplicate sample for LAMP testing were included in analysis (see Appendices: 1-All data, 2-Matched Clinical Data).

Standard measurements for statistical analysis were used to calculate:

Sensitivity (SE) or true positive rate (TPR)

SE = True Positive (TP) / (True Positive (TP) + False Negative (FN))

Specificity (SP) or true negative rate (TNR) = N targets – TP – False Positive (FP) - FN

SP = True Negative / (TN + FP)

Precision or positive predictive value (PPV)

PPV = TP / (TP + FP)

Negative predictive value (NPV)

NPV = TN / (TN + FN)

Sensitivity for each organism was determined based on positive findings on In-Dx in comparison to hospital methods abstracted from clinical results.

### Thermocycler SYTO-82 Reaction Analysis

Threshold cycle (C_t_) was calculated as time in which signal increased 0.01 arbitrary units above the original signal for real-time analysis. One cycle is completed every 53 s. Overall, results were considered positive if two out of three wells exhibited amplification.

### Eriochrome Black T (EBT) Reaction Analysis

The threshold for identification of positive amplification was positive color change from purple to blue in at least two out of three wells for each gene target. Results were determined by visual discrimination by one or more non-color blind observers who were blinded to the targets in each well. All plates were read immediately after reaction completion.

## Author Contributions

BE: study proposal, study design, laboratory methodology development, patient enrollment, sample collection, data analysis, and manuscript preparation. ZL: laboratory methodology development, laboratory sample processing, and data analysis. RS: study design and laboratory methodology development. MN: laboratory methodology development, laboratory sample processing, and data analysis. MW: laboratory methodology development. TJ: study design. TS: laboratory methodology development. TK: study design and laboratory methodology development. WK: study design, laboratory methodology development, and clinical microbial data analysis. JT: study design and laboratory methodology development. SH: study design and laboratory methodology development. MH: study design, clinical patient and sample coordination, patient enrollment, clinical sample collection, and clinical data analysis.

## Conflict of Interest Statement

The authors declare that the research was conducted in the absence of any commercial or financial relationships that could be construed as a potential conflict of interest.

## Footnotes

1 https://primerexplorer.jp/e/

## References

[B1] Altschul S. F. Gish W. Miller W. Myers E. W. Lipman D. J. (1990). Basic local alignment search tool. *J. Mol. Biol.* 215 403–410. 10.1016/S0022-2836(05)80360-2 2231712

[B2] Angus D. C. Linde-Zwirble W. T. Lidicker J. Clermont G. Carcillo J. Pinsky M. R. (2001). Epidemiology of severe sepsis in the United States: Analysis of incidence, outcome, and associated costs of care. *Critical Care Medicine* 29 1303–1310. 10.1097/00003246-200107000-00002 11445675

[B3] Avolio M. Diamante P. Zamparo S. Modolo M. L. Grosso S. Zigante P. (2010). Molecular identification of bloodstream pathogens in patients presenting to the emergency department with suspected sepsis. *Shock* 34 27–30. 10.1097/SHK.0b013e3181d49299 20090568

[B4] Becker K. Heilmann C. Peters G. (2014). Coagulase-negative staphylococci. *Clin Microbiol Rev* 27 870–926. 10.1128/CMR.00109-13 25278577 PMC4187637

[B5] Dugan L. Bearinger J. Hinckley A. Strout C. Souza B. (2012). Detection of Bacillus anthracis from spores and cells by loop-mediated isothermal amplification without sample preparation. *J Microbiol Methods* 90 280–284. 10.1016/j.mimet.2012.05.022 22677603

[B6] Geojith G. Dhanasekaran S. Chandran S. P. Kenneth J. (2011). Efficacy of loop mediated isothermal amplification (LAMP) assay for the laboratory identification of Mycobacterium tuberculosis isolates in a resource limited setting. *J Microbiol Methods* 84 71–73. 10.1016/j.mimet.2010.10.015 21047534

[B7] Jevtuševskaja J. Uusna J. Andresen L. Krõlov K. Laanpere M. Grellier T. (2016). Combination with antimicrobial peptide lyses improves loop-mediated isothermal amplification based method for Chlamydia trachomatis detection directly in urine sample. *BMC Infect Dis* 16:329. 10.1186/s12879-016-1674-0 27412444 PMC4944247

[B8] Johansson K. Karlsson H. Norén T. (2016). Clostridium difficile infection diagnostics - evaluation of the C. DIFF Quik Chek Complete assay, a rapid enzyme immunoassay for detection of toxigenic C. difficile in clinical stool samples. *Apmis* 124 1016–1020. 10.1111/apm.12595 27651167

[B9] Johnston A. N. B. Park J. Doi S. A. Sharman V. Clark J. Robinson J. (2017). Effect of Immediate Administration of Antibiotics in Patients With Sepsis in Tertiary Care: A Systematic Review and Meta-analysis. *Clin Ther* 39 190–202e196. 10.1016/j.clinthera.2016.12.003 28062114

[B10] Kimura Y. de Hoon M. J. Aoki S. Ishizu Y. Kawai Y. Kogo Y. (2011). Optimization of turn-back primers in isothermal amplification. *Nucleic Acids Res* 39 e59. 10.1093/nar/gkr041 21310714 PMC3089485

[B11] Li D. Yang M. Hu J. Zhang J. Liu R. Gu X. (2009). Antibiotic-resistance profile in environmental bacteria isolated from penicillin production wastewater treatment plant and the receiving river. *Environ Microbiol* 11 1506–1517. 10.1111/j.1462-2920.2009.01878.x 20400569 PMC2876458

[B12] Loo J. F. Kwok H. C. Leung C. C. Wu S. Y. Law I. L. Cheung Y. K. (2016). Sample-to-answer on molecular diagnosis of bacterial infection using integrated lab–on–a–disc. *Biosens Bioelectron* 93 212–219. 10.1016/j.bios.2016.09.001 27660018

[B13] Misawa Y. Yoshida A. Saito R. Yoshida H. Okuzumi K. Ito N. (2007). Application of loop-mediated isothermal amplification technique to rapid and direct detection of methicillin-resistant *Staphylococcus aureus* (MRSA) in blood cultures. *J Infect Chemother* 13 134–140. 10.1007/s10156-007-0508-9 17593498

[B14] Notomi T. Okayama H. Masubuchi H. Yonekawa T. Watanabe K. Amino N. (2000). Loop-mediated isothermal amplification of DNA. *Nucleic Acids Res* 28 E63 10.1093/nar/28.12.e63 PMC102748 10871386

[B15] Oh S. J. Park B. H. Jung J. H. Choi G. Lee D. C. Kim D. H. (2016). Centrifugal loop-mediated isothermal amplification microdevice for rapid, multiplex and colorimetric foodborne pathogen detection. *Biosens Bioelectron* 75 293–300. 10.1016/j.bios.2015.08.052 26322592

[B16] Poon L. L. Wong B. W. Ma E. H. Chan K. H. Chow L. M. Abeyewickreme W. (2006). Sensitive and inexpensive molecular test for falciparum malaria: detecting Plasmodium falciparum DNA directly from heat-treated blood by loop-mediated isothermal amplification. *Clin Chem* 52 303–306. 10.1373/clinchem.2005.057901 16339303

[B17] Rivers E. P. Katranji M. Jaehne K. A. Brown S. Abou Dagher G Cannon C. (2012). Early interventions in severe sepsis and septic shock: a review of the evidence one decade later. *Minerva Anestesiol* 78 712–724. 22447123

[B18] Rossi C. C. Souza-Silva T. Araújo-Alves A. V. Giambiagi-deMarval M. (2017). CRISPR-Cas Systems Features and the Gene-Reservoir Role of Coagulase-Negative Staphylococci. *Front Microbiol* 8:1545. 10.3389/fmicb.2017.01545 28861060 PMC5559504

[B19] Stedtfeld R. D. Liu Y. C. Stedtfeld T. M. Kostic T. Kronlein M. Srivannavit O. (2015). Static self-directed sample dispensing into a series of reaction wells on a microfluidic card for parallel genetic detection of microbial pathogens. *Biomed Microdevices* 17 89. 10.1007/s10544-015-9994-1 26260693 PMC4531140

[B20] Tourlousse D. M. Ahmad F. Stedtfeld R. D. Seyrig G. Tiedje J. M. Hashsham S. A. (2012). A polymer microfluidic chip for quantitative detection of multiple water- and foodborne pathogens using real-time fluorogenic loop-mediated isothermal amplification. *Biomed Microdevices* 14 769–778. 10.1007/s10544-012-9658-3 22566273

[B21] Tsalik E. L. Jones D. Nicholson B. Waring L. Liesenfeld O. Park L. P. (2010). Multiplex PCR to diagnose bloodstream infections in patients admitted from the emergency department with sepsis. *J Clin Microbiol* 48 26–33. 10.1128/JCM.01447-09 19846634 PMC2812289

[B22] van der Zee A. Roorda L. Bosman G. Ossewaarde J. M. (2016). Molecular Diagnosis of Urinary Tract Infections by Semi-Quantitative Detection of Uropathogens in a Routine Clinical Hospital Setting. *PLOS One* 11:e0150755. 10.1371/journal.pone.0150755 26954694 PMC4783162

[B23] van Oort P. M. Nijsen T. Weda H. Knobel H. Dark P. Felton T. (2017). BreathDx - molecular analysis of exhaled breath as a diagnostic test for ventilator-associated pneumonia: protocol for a European multicentre observational study. *BMC Pulm Med* 17:1. 10.1186/s12890-016-0353-7 28049457 PMC5210294

[B24] Wang X. Yin F. Bi Y. Cheng G. Li J. Hou L. (2016). Rapid and sensitive detection of Zika virus by reverse transcription loop-mediated isothermal amplification. *J Virol Methods* 238 86–93. 10.1016/j.jviromet.2016.10.010 27793644

[B25] Williams M. R. Stedtfeld R. D. Waseem H. Stedtfeld T. Upham B. Khalife W. (2017). Implications of direct amplification for measuring antimicrobial resistance using point-of-care devices. *Anal. Methods* 9 1229–1241. 10.1039/c6ay03405e PMC5898395 29657581

[B26] Yan M. Li W. Zhou Z. Peng H. Luo Z. Xu L. (2016). Direct detection of various pathogens by loop-mediated isothermal amplification assays on bacterial culture and bacterial colony. *Microb Pathog* 102 1–7. 10.1016/j.micpath.2016.10.025 27836764

