# Supplementary material for: Evaluation of Nucleic Acid Isothermal Amplification Methods for Human Clinical Microbial Infection Detection
Source: Front Microbiol. 2017 Dec 12;8:2211. doi: 10.3389/fmicb.2017.02211 (PMC5732957; doi:10.3389/fmicb.2017.02211)
Supplement: Supplementary file 1 [file Presentation_1.PPTX]

## Slide 1
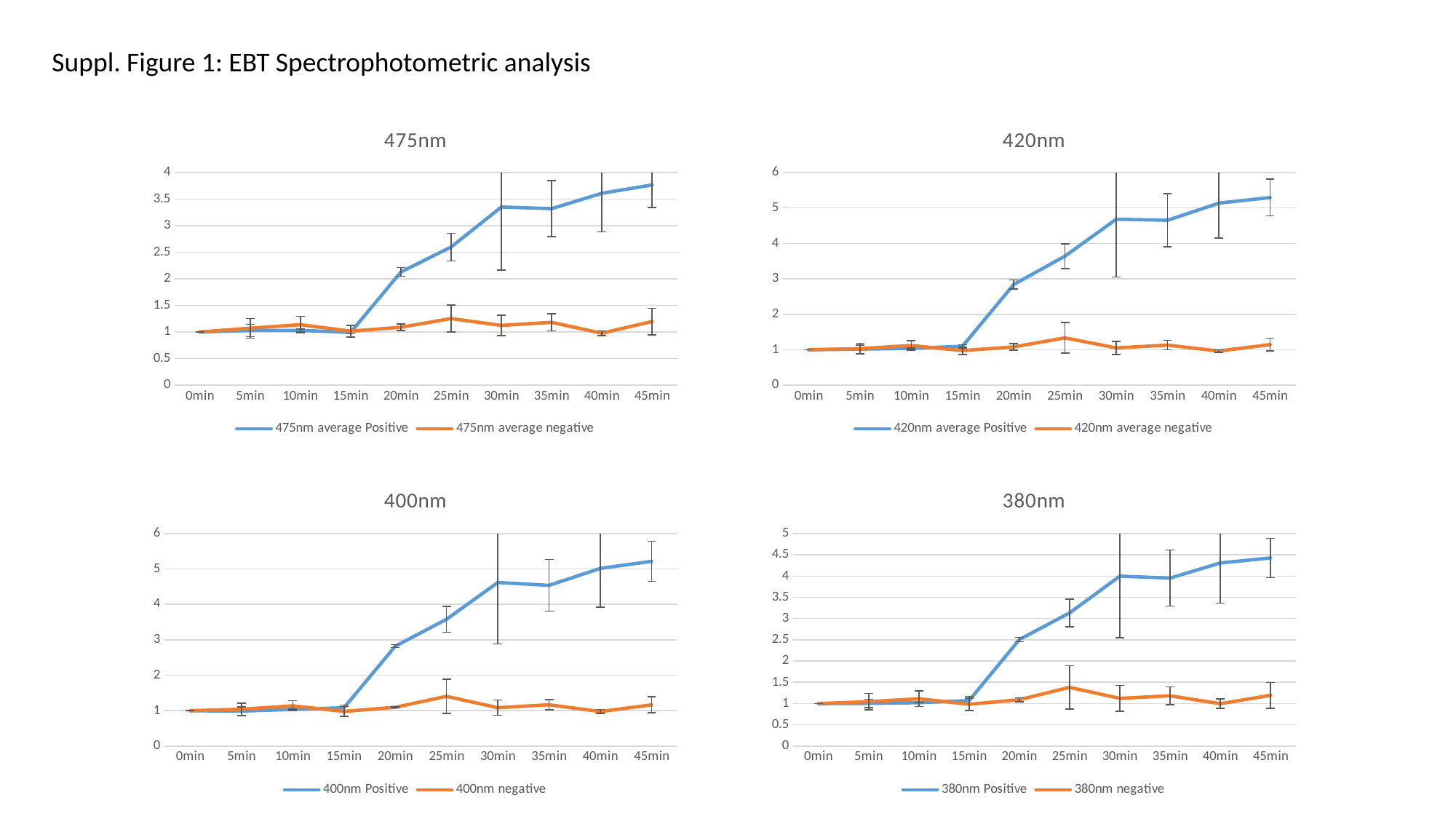

Suppl. Figure 1: EBT Spectrophotometric analysis
### Chart: 475nm
| Category | 475nm average | 475nm average |
|---|---|---|
| 0min | 1.0 | 1.0 |
| 5min | 1.029950669485554 | 1.072198333067898 |
| 10min | 1.030655391120508 | 1.137649307214524 |
| 15min | 0.992424242424242 | 1.016032276901842 |
| 20min | 2.13002114164905 | 1.089791368052238 |
| 25min | 2.599189570119803 | 1.252672930933801 |
| 30min | 3.35183227625088 | 1.124133354568137 |
| 35min | 3.321353065539113 | 1.181684981684982 |
| 40min | 3.609760394644116 | 0.977284068588416 |
| 45min | 3.766913319238901 | 1.197717258586824 |
### Chart: 420nm
| Category | 420nm average | 420nm average |
|---|---|---|
| 0min | 1.0 | 1.0 |
| 5min | 1.017316017316017 | 1.029934210526316 |
| 10min | 1.038952550717257 | 1.12013888888889 |
| 15min | 1.098684322213734 | 0.975767543859649 |
| 20min | 2.841931924284863 | 1.080043859649123 |
| 25min | 3.641592394533571 | 1.335343567251462 |
| 30min | 4.683855360325944 | 1.052156432748538 |
| 35min | 4.65450301332654 | 1.130811403508772 |
| 40min | 5.137619896443425 | 0.963267543859649 |
| 45min | 5.295068330362447 | 1.144005847953217 |
### Chart: 400nm
| Category | 400nm | 400nm |
|---|---|---|
| 0min | 1.0 | 1.0 |
| 5min | 0.988791423001949 | 1.041075917546506 |
| 10min | 1.03604920710184 | 1.136701860231272 |
| 15min | 1.082345503398135 | 0.979587732528909 |
| 20min | 2.8202281228597 | 1.09693313222725 |
| 25min | 3.579500553184764 | 1.404172951231775 |
| 30min | 4.617867867867866 | 1.085470085470085 |
| 35min | 4.538024866972234 | 1.167320261437909 |
| 40min | 5.016345292661082 | 0.975666163901458 |
| 45min | 5.215544491860282 | 1.165258924082454 |
### Chart: 380nm
| Category | 380nm | 380nm |
|---|---|---|
| 0min | 1.0 | 1.0 |
| 5min | 1.006475485661425 | 1.046064751481988 |
| 10min | 1.021430773974715 | 1.11450980392157 |
| 15min | 1.072155411655874 | 0.98359325125399 |
| 20min | 2.507246376811594 | 1.092275421796626 |
| 25min | 3.133055812519272 | 1.382289101687186 |
| 30min | 3.997687326549491 | 1.123529411764706 |
| 35min | 3.951742213999382 | 1.18388508891929 |
| 40min | 4.307739747147701 | 0.99813041495668 |
| 45min | 4.424761023743447 | 1.195230278157775 |

## Slide 2
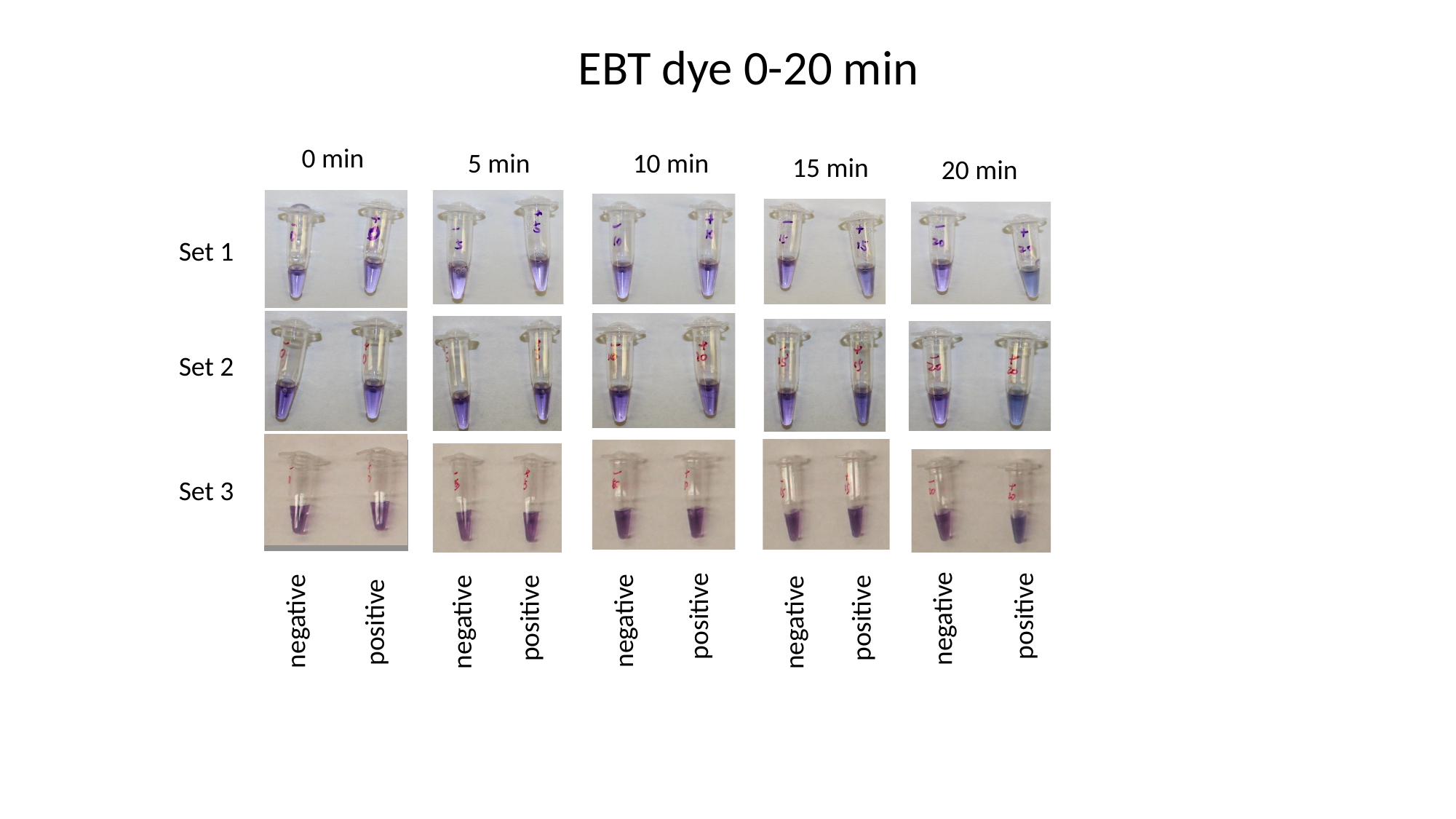

EBT dye 0-20 min
0 min
5 min
10 min
15 min
20 min
Set 1
Set 2
Set 3
positive
positive
positive
positive
negative
negative
negative
negative
positive
negative

## Slide 3
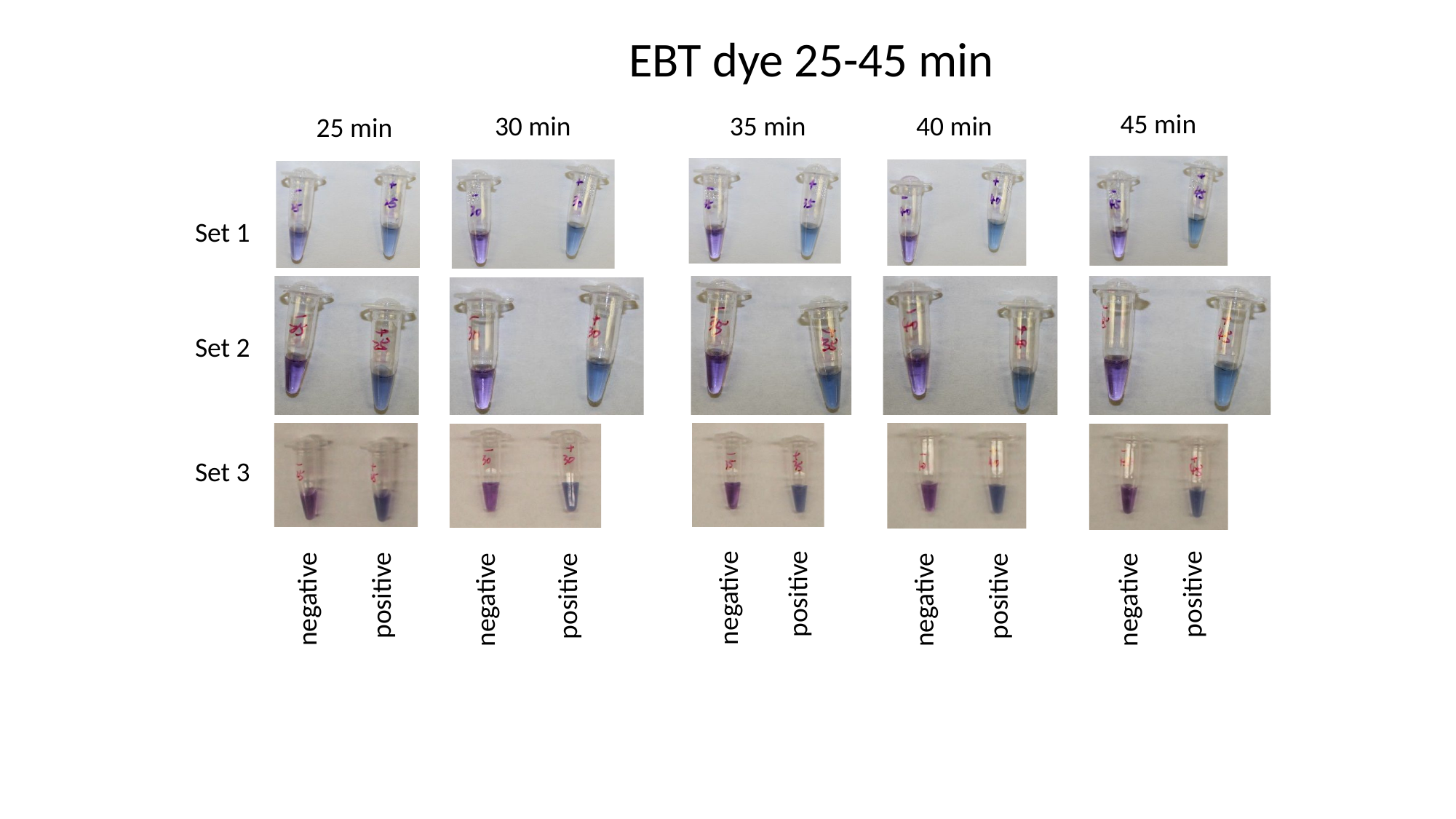

EBT dye 25-45 min
45 min
35 min
40 min
30 min
25 min
Set 1
Set 2
Set 3
positive
positive
positive
positive
positive
negative
negative
negative
negative
negative

## Slide 4
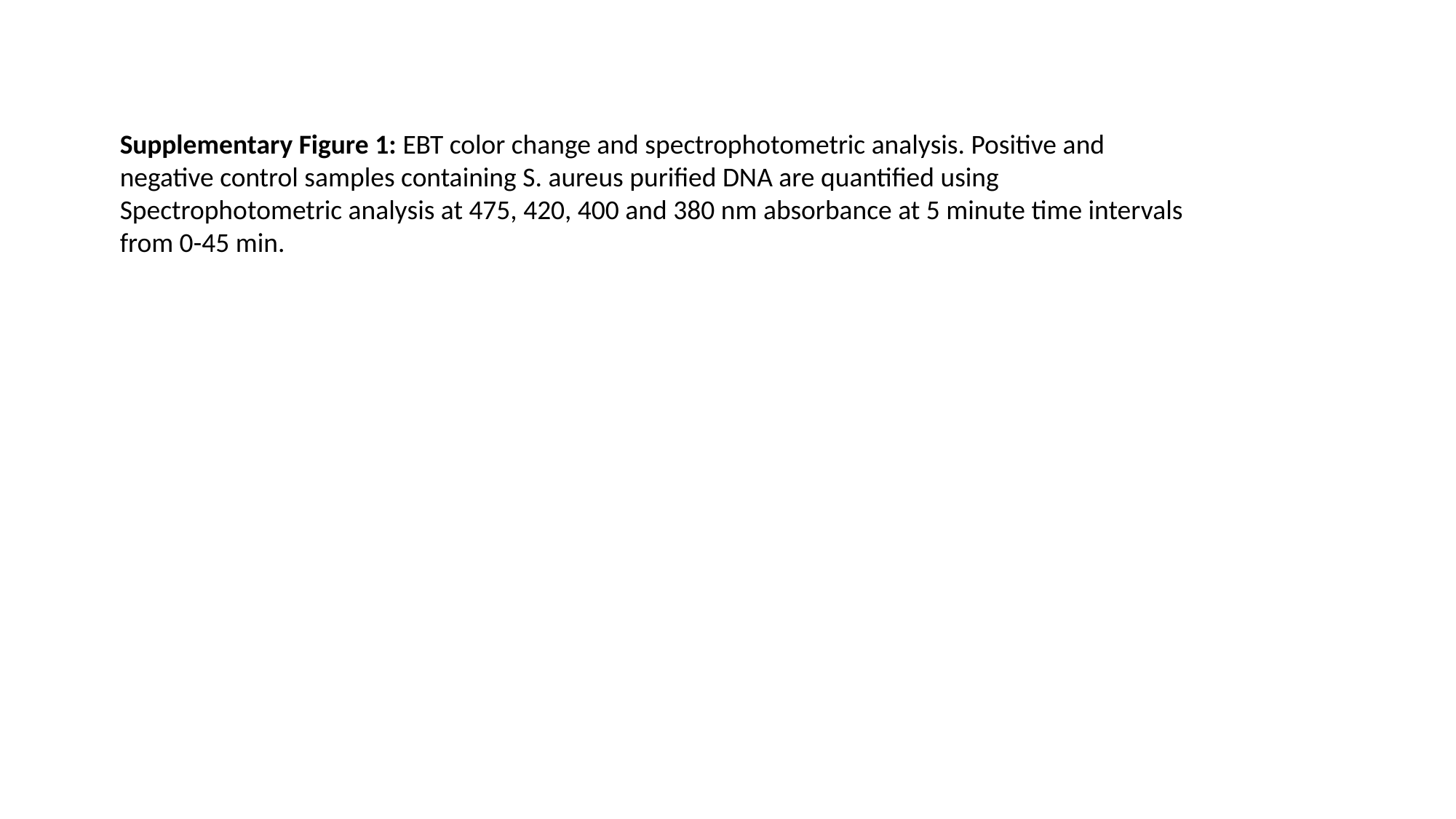

Supplementary Figure 1: EBT color change and spectrophotometric analysis. Positive and negative control samples containing S. aureus purified DNA are quantified using Spectrophotometric analysis at 475, 420, 400 and 380 nm absorbance at 5 minute time intervals from 0-45 min.
